# HISEA: HIerarchical SEed Aligner for PacBio data

**DOI:** 10.1186/s12859-017-1953-9

**Published:** 2017-12-19

**Authors:** Nilesh Khiste, Lucian Ilie

**Affiliations:** Department of Computer Science, University of Western Ontario, LondonOntario, N6A 5B7 Canada

**Keywords:** PacBio sequencing, Read aligner, Read overlapper, Genome assembly

## Abstract

**Background:**

The next generation sequencing (NGS) techniques have been around for over a decade. Many of their fundamental applications rely on the ability to compute good genome assemblies. As the technology evolves, the assembly algorithms and tools have to continuously adjust and improve. The currently dominant technology of Illumina produces reads that are too short to bridge many repeats, setting limits on what can be successfully assembled. The emerging SMRT (Single Molecule, Real-Time) sequencing technique from Pacific Biosciences produces uniform coverage and long reads of length up to sixty thousand base pairs, enabling significantly better genome assemblies. However, SMRT reads are much more expensive and have a much higher error rate than Illumina’s – around 10-15% – mostly due to indels. New algorithms are very much needed to take advantage of the long reads while mitigating the effect of high error rate and lowering the required coverage.

**Methods:**

An essential step in assembling SMRT data is the detection of alignments, or overlaps, between reads. High error rate and very long reads make this a much more challenging problem than for Illumina data. We present a new pairwise read aligner, or overlapper, HISEA (Hierarchical SEed Aligner) for SMRT sequencing data. HISEA uses a novel two-step k-mer search, employing consistent clustering, k-mer filtering, and read alignment extension.

**Results:**

We compare HISEA against several state-of-the-art programs – BLASR, DALIGNER, GraphMap, MHAP, and Minimap – on real datasets from five organisms. We compare their sensitivity, precision, specificity, F1-score, as well as time and memory usage. We also introduce a new, more precise, evaluation method. Finally, we compare the two leading programs, MHAP and HISEA, for their genome assembly performance in the Canu pipeline.

**Discussion:**

Our algorithm has the best alignment detection sensitivity among all programs for SMRT data, significantly higher than the current best. The currently best assembler for SMRT data is the Canu program which uses the MHAP aligner in its pipeline. We have incorporated our new HISEA aligner in the Canu pipeline and benchmarked it against the best pipeline for multiple datasets at two relevant coverage levels: 30x and 50x. Our assemblies are better than those using MHAP for both coverage levels. Moreover, Canu+HISEA assemblies for 30x coverage are comparable with Canu+MHAP assemblies for 50x coverage, while being faster and cheaper.

**Conclusions:**

The HISEA algorithm produces alignments with highest sensitivity compared with the current state-of-the-art algorithms. Integrated in the Canu pipeline, currently the best for assembling PacBio data, it produces better assemblies than Canu+MHAP.

**Electronic supplementary material:**

The online version of this article (doi:10.1186/s12859-017-1953-9) contains supplementary material, which is available to authorized users.

## Background

*De novo* genome assembly is the problem of reconstructing the entire genome of an organism from sequencing reads without using a reference genome. The high throughput NGS technologies produce short reads, of few hundred base pairs, which are much smaller than most of the repeated regions in microbial and eukaryotic genomes. The repeated regions that are longer than read length pose serious challenges to the genome assembly algorithm. This imbalance of read versus repeat length increases the complexity and processing requirements of the assembly algorithm. This is the reason many assemblies using NGS data are fragmented and incomplete [1], and often not useful for downstream analysis.

The advent of SMRT sequencing technology from Pacific Biosciences has encouraged researchers to look into the genome assembly problem from a fresh perspective. The long reads spanning across many repeated regions enable the production of significantly better assemblies. The SMRT technology is also less biased [2] than previous NGS technologies. However, two important drawbacks of SMRT sequencing are high error rate, of 10-15%, and high cost. For comparison, the dominant technology of Illumina has up to 100 times lower error rate and is over 100 times cheaper in terms of cost per Gbp [3]. On the positive side, it has been found that the errors are random and it is possible to correct them algorithmically [4] by increasing the coverage of sequencing data. Thus, SMRT sequencing makes it possible to produce more continuous and higher quality genome assemblies than what has been achieved with previous technologies.

In most of the published SMRT genome assembly pipelines [5–7], a critical step is finding all-vs-all raw read alignments. The outcome of this step can have a large impact on the processing of subsequent steps and the overall outcome of the assembly pipeline. It is therefore essential to use a highly sensitive aligner. We present a new long read aligner, HISEA, which is much more sensitive than all existing ones. We compared the sensitivity of our aligner with BLASR [8], DALIGNER [9], GraphMap [10], MHAP [11], and MiniMap [5]. Note that we use the terms “alignment” and “overlap” interchangeably.

The comparatively high cost of SMRT sequencing has prevented its widespread use. It is very expensive to sequence large genomes with high coverage using SMRT technology, therefore it is still beyond the reach of many research labs. Recently, Koren et al. [6] showed that their Canu assembler can generate assemblies using only 20x coverage that are comparable with 150x coverage hybrid assemblies generated with SPAdes [12]. It has also shown that it can achieve maximum assembly continuity around 50x coverage. As indicated by Koren et al. [6], Canu pipeline is currently the best. It uses the MHAP aligner [11] and therefore we incorporated HISEA in this assembly pipeline, in place of MHAP. We have compared the two pipelines, Canu+MHAP and Canu+HISEA for five organisms, *E.coli*, *S.cerevisiae*, *C.elegans*, *A.thaliana*, and *D.melanogaster* at two coverage levels: 30x and 50x. The pipeline using HISEA is shown to produce better assemblies for both coverage levels. Moreover, the Canu+HISEA assemblies for 30x coverage are comparable with those of Canu+MHAP for 50x coverage.

Our HISEA software is implemented in C++ and OpenMP and its source code is freely available. It can be used as a stand alone aligner or as an all-vs-all read aligner in other assembly pipelines. We have tested it in the Canu [6] assembly pipeline and the modified pipeline source code is also freely available for download.

## Methods

### The HISEA algorithm

Let *Σ*={*A*,*C*,*G*,*T*} be the DNA alphabet; *Σ* ^∗^ is the set of all DNA sequences, that is, all finite strings over *Σ*. Our setup assumes two sets of reads: the set of reference reads, *R*={*r*_1_,*r*_2_,...,*r*_*n*_}⊂*Σ* ^∗^, and the set of query reads, *Q*={*q*_1_,*q*_2_,...,*q*_*m*_}⊂*Σ* ^∗^. A *k*-mer is a string of length *k* over *Σ*.

#### Storing reads and hashing the reference set

Each read *r*_*i*_ is encoded using 2 bits per nucleotide and stored as an array of unsigned 64-bit integers, that is, as blocks of 32 nucleotides. The reverse complement of *r* is stored in the same array and it starts at the next unsigned 64-bit integer. A precomputed 16-bit reverse complement array of all possible values is used to quickly compute the reverse complement of reads.

All *k*-mers that occur in reads of *R* are quickly computed using bitwise operations and bit masking and stored in a hash table using double hashing technique. In the hash table, each entry stores the value of the *k*-mer and a pointer to another hash table which stores the set of read ids *r*_*j*_, and positions within *r*_*j*_, where this *k*-mer occurs. Any *k*-mer which occurs more than MAX_KMER_COUNT times is ignored. The MAX_KMER_COUNT is a user configurable parameter with a default value of 10000. Similarly, *k*-mers appearing in low count can be ignored. These *k*-mers do not impact the alignment and ignoring them speeds up the alignment process. The default value for low count *k*-mers is 2 and it can be controlled by a user configurable parameter.

#### Searching the query set

The *k*-mers occurring in the query read set *Q* are not stored; they are quickly computed as needed using bit operations. Then they are efficiently searched for in the hash table built for the set *R*. Every time a matching *k*-mer is found in the hash table, the corresponding reference read id and its position is recorded. Note that the reads in the query set are only searched in forward direction.

#### Clustering and filtering

For a given query *q*∈*Q* and a reference read *r*∈*R*, the reference read direction and all matching *k*-mer positions are stored in the previous step. For a pair of reads (*q*, *r*), further processing is considered either in forward or reverse direction of *r*. The decision is taken based on the read direction of *r* which has higher number of matching *k*-mers.

**Fig. 1:**
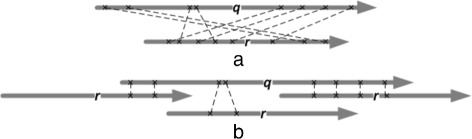
All *k*-mer matches between reads *q* and *r* before (**a**) and after (**b**) clustering

The next step is to perform clustering of all the matching *k*-mers. Clustering is an essential step in identifying the best alignment out of multiple possible alignments. Our algorithm reports only the best alignment between a pair of reads. Figure 1 shows an example of all *k*-mer matches between read *q* and read *r* before and after clustering. The example shown here is one simple case; in reality many complex cases are possible where clustering is essential. The initial matches can have contradictory information, such as the ones in Fig. 1 a, and the clustering phase involves collecting together consistent matches. A *consistent* set of *k*-mer matches is defined as a set of all *k*-mer matches arranged in ascending order of their positions and are equidistant from neighboring *k*-mer matches within defined threshold. The threshold is governed by a global parameter *m* *a* *x* *S* *h* *i* *f* *t*. The parameter *m* *a* *x* *S* *h* *i* *f* *t* is a user configurable parameter that accommodates the *indel* errors during *k*-mer matching, clustering and extension algorithms. The default value of this parameter has been experimentally determined to be 0.2 (or 20%). Figure 1 b shows the set of *k*-mers as divided into three consistent groups. It can be seen from the diagram that the rightmost cluster of *k*-mers is expected to produce the best alignment.

Algorithm 2.1 gives the details of the clustering algorithm. The input to the algorithm is an array *V* which contains all *k*-mer matches for a pair of reads (*q*, *r*). The input *k*-mer matches in *V* are sorted beforehand, first by query read positions and then by reference read positions. If the clustering algorithm fails to produce any meaningful clusters, we reverse the sort order i.e. first sort by reference read positions and then by query read positions and retry the algorithm. The algorithm uses two global parameters, *k* *m* *e* *r* *S* *i* *z* *e* and *m* *a* *x* *S* *h* *i* *f* *t*. The parameter *k* *m* *e* *r* *S* *i* *z* *e* is the size of the *k*-mers used for the initial hashing. The parameter *m* *a* *x* *S* *h* *i* *f* *t* is defined previously. The output of the clustering algorithm is a set of matches, *C* *l* *u* *s* *t* *e* *r* *A* *r* *r* *a* *y*, segregated in groups such that each group has a consistent set of *k*-mers. Note that the first two values in *C* *l* *u* *s* *t* *e* *r* *A* *r* *r* *a* *y* store the left and right *k*-mer positions in *V* for that cluster. The third and fourth values are the number of matching bps and *k*-mer hit counts respectively.

**Fig. 2:**
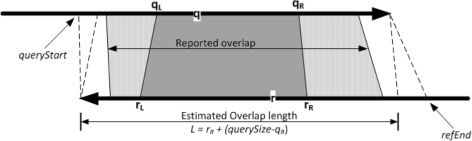
Computing the alignment. The dark grey region contains all *k*-mer matches and is extended by the light grey ones using *k* ^′^-mer matches



From the output of Algorithm 2.1, the cluster with the maximum number of matching base pairs is selected for further processing. The expected number of *k*-mer matches is estimated with the help of *k*-mer bounds in read *q* and read *r*; see Fig. 2. The leftmost and rightmost query *k*-mers start and end at positions *q*_*L*_ and *q*_*R*_, respectively. Similarly, the corresponding positions in the reference read are *r*_*L*_ and *r*_*R*_. The alignment length is *L*=*r*_*R*_+*q* *u* *e* *r* *y* *S* *i* *z* *e*−*q*_*R*_. The number of *k*-mer hits in the overlapping region is approximated as a binomial distribution with probability *p*=(1−*e*)^2*k*^ and *L* trials. Overlaps that have fewer *k*-mer matches than three standard deviations below the mean, that is, less than $\mu - 3\sigma = Lp - 3\sqrt {Lp(1-p)}$, are eliminated as having too low similarity. This procedure is employed several times during different steps of the algorithm and will be referred to as the *μ*−3*σ* *criterion*.

#### Computing alignments

The alignment between the two given reads starts as the shortest interval that contains all *k*-mer matches, shown in dark grey in Fig. 2. This region is extended using a smaller seed, that is, using *k* ^′^-mer matches, for some *k* ^′^<*k*. The default values are *k*=16 and *k* ^′^=12. These values have been determined experimentally to produce reasonably good results for most datasets. Note that MHAP uses 16-mers as well.

The first step is to compute the maximum bounds of the alignment considering the maximum amount of allowable indels in the overlapping region. This is given by the user configurable parameter *m* *a* *x* *S* *h* *i* *f* *t* we mentioned above. As an example, for the situation depicted in Fig. 2, we set the maximum bounds for read *q* and read *r* as (*q* *u* *e* *r* *y* *S* *t* *a* *r* *t*,*q* *u* *e* *r* *y* *S* *i* *z* *e*) and (0,*r* *e* *f* *E* *n* *d*) respectively (see Fig. 2) where:

$$\begin{array}{rcl} {queryStart} &=& q_{L} - (1+{maxShift})r_{L}\\ {refEnd} &=& r_{R} + (1+{maxShift})({querySize} - q_{R}) \end{array} $$

Then, all *k* ^′^-mer matches within these bounds are computed as done previously for *k*-mers. These matches are used to extend the alignment we have computed so far; in Fig. 2, the dark grey region is extended by the light grey ones on both sides. Each *k* ^′^-mer match is added if together with the ones already added they satisfy the *μ*−3*σ* criterion described above. The structure of the extension step is given in Algorithm 2.2. The input bounds are either (*q*_*L*_,*r*_*L*_) or (*q*_*R*_,*r*_*R*_). The extension is performed as long as *k* ^′^-mer matches exist that satisfy the *μ*−3*σ* criterion.



Finally, all the *k* ^′^-mers within the initial region – dark grey colour in Fig. 2 – are computed. Note also that the process is now guided by the original *k*-mers and therefore the clustering step is not required. The *μ*−3*σ* criterion is applied once more to the total number of *k* ^′^-mer matches for the entire overlap (light and dark grey); if the condition is satisfied, then the reads are considered to be overlapping and the alignment is reported.

Note that HISEA computes only the alignment boundaries, not the actual alignments. The same is true for other programs, such as MHAP [11], Minimap [5], and GraphMap [10]. Once identified, the alignments can be computed by dynamic programming; we avoid this step as it is very time consuming and not necessary for assembly, which is the goal of HISEA.

### Alignment evaluation procedures

The *EstimateROC* utility estimates the sensitivity, specificity, and precision for the alignments reported. The original *EstimateROC* utility of Berlin et al. [11] relies heavily on BLASR mappings for the verification of reported alignments. This is not the most accurate procedure since BLASR can make errors. Ideally, each alignment needs to be verified against the optimally computed alignment using the Smith-Waterman dynamic programming algorithm [13]. We modified the functions estimating sensitivity, specificity, and precision accordingly.

The modified function *ComputeDP* first computes an optimal alignment, *A*_opt_, between two reads using Smith-Waterman dynamic programming algorithm; it ensures that this is a good alignment. Then, assuming the program reported an alignment, *A*_rep_, for these two reads, it compares the length, direction, and bounds of the alignment reported by the program with those of the optimal alignment. This is essential since the program could report a very different alignment between the same reads and that should not be considered correct. The use of an optimal alignment algorithm increases the accuracy of evaluation.

The three functions used for evaluation, *EstimateSensitivity, EstimateSpecificity*, and *EstimatePrecision* are modified to correspond with our new *ComputeDP* function. The details are given in pseudo code below; see Algorithms 3.3-3.6.

Note that our evaluation is more accurate than the one of Berlin et al. [11] and all programs exhibit an decline in performance. The Results section contains a comparison of several evaluation procedures.









## Results

### Datasets

All the datasets have been downloaded from Pacific Biosciences DevNet Datasets (https://github.com/PacificBiosciences/DevNet/wiki/Datasets). The datasets used for this evaluation are given in Table 1. Details are provided in the Additional file 1.

**Table 1:** SMRT datasets used in for evaluation

Genome	Reference	Coverage	Chemistry	Genome size
	number			(Mbp)
*E.coli*	NC_000913	85x	P5C3	4.64
*S.cerevisiae*	NC_001133.9	117x	P4C2	12.16
*C.elegans*	WS222	80x	P6C4	100.2
*A.thaliana*	TAIR10	110x	P4C2	134.6
*D.melanogaster*	Ref v5	90x	P5C3	129.7

The tests were performed on a DELL PowerEdge R620 computer with 12 cores Intel Xeon at 2.0 GHz and 256 GB of RAM, running Linux Red Hat, CentOS 6.3.

### Competing programs

We evaluated the performance of HISEA against the currently best programs for PacBio read alignment: BLASR [8], DALIGNER [9], GraphMap [10], MHAP [11], and Minimap [5]. We then assessed the performance of HISEA for assembling PacBio data by including HISEA in the Canu assembly pipeline [6] and comparing it with the Canu assembly using MHAP as the aligner.

The programs were run according to their own developers’ suggestions or better, as follows. Minimap and DALIGNER were run as suggested by the developers. BLASR was run according to what the MHAP paper claimed to be the best choice of parameters. This is clearly better than the default parameters of BLASR. GraphMap was run with default parameters as the only choice in overlapping mode. MHAP was run with default parameters, except the number of hashes, which was set to 1256, instead of the default 512, for increased sensitivity. Minimap was run with window size 5 (default is 10), as recommended by the designers. HISEA was run with default parameters.

The Additional file 1 contains all the details concerning the versions used, download websites, and command lines.

### Alignment comparison

The first tests we performed, as done also by Berlin et al. [11], use subdatasets of 1Gbp randomly sampled from the initial datasets; for the two smallest genomes, *E.coli* and *S.cerevisiae*, full datasets are used since they are close to 1Gbp with the given coverage. The sensitivity, specificity, and precision values for all five programs are given in Table 2. They were computed using the *EstimateSensitivity, EstimateSpecificity*, and *EstimatePrecision* procedures that we described in the Methods section.

**Table 2:** Comparison for the 1Gbp datasets (coverage levels in parentheses)

Sensitivity, specificity, precision, and *F*_1_-score are given as percentages; A dash mean that the program crashed with segmentation fault. The best values are shown in bold. The bottom of the table shows the average values, each computed from the five corresponding values in the table

Similarly to MHAP [11] evaluation parameters for *EstimateROC*, we use minimum alignment length 2000 bps and 50,000 trials. The other mandatory inputs to *EstimateROC* are the reference genome, the reads and the mapping of the reads to the reference. The mapping of the reads to the reference is computed using the BLASR program.

HISEA has clearly the highest sensitivity, over 16% higher, on the average, than the second best program, MHAP. The specificity is high for all programs. Minimap has the highest specificity but low sensitivity. BLASR has the highest precision but, again, low sensitivity. HISEA is second for precision, not far from BLASR. To better compare the performance with respect to sensitivity and precision, we have computed the *F*_1_-scores, also shown in Table 2. The *F*_1_-score for HISEA is much higher than all the other programs, with DALIGNER and MHAP following 18% and 19% behind. Next are BLASR and Minimap and last comes GraphMap with a very low score.

The time and memory comparison for the same 1Gbp datasets is presented in Table 3. Minimap and GraphMap are clearly the fastest and BLASR the slowest. HISEA is in the middle, behind MHAP and DALINER. Space-wise, Minimap is again the best, followed closely by BLASR, and at some distance by HISEA and GraphMap. MHAP and DALIGNER used the most memory. MHAP is implemented in JAVA which generally requires more memory. The java command-line parameter -Xmx is used to set the maximum heap size for MHAP stand alone invocation. The default maximum java heap size depends on the platform and the amount of memory in the system. For our systems, the default was not sufficient to perform the tests. We set -Xmx parameter to 200G which was sufficient for all tests but it does not capture true overlapper memory for MHAP. The reported memory usage for MHAP consists of the overlapper memory and the memory required for Java Virtual Machine environment.

**Table 3:** Time and memory comparison for the 1Gbp datasets

Genome	Time (h)	BLASR	DALIGNER	GraphMap	MHAP	Minimap	HISEA
	Memory (GB)						
E.coli	Time	113.0	3.0	0.3	3.0	**0.1**	4.0
	Memory	**7.1**	124.6	42.3	210.0	8.8	25.5
S.cerevisiae	Time	283.2	–	0.6	10.6	**0.3**	23.5
	Memory	**13.3**	–	71.0	210.0	15.1	56.5
C.elegans	Time	333.6	4.1	0.6	4.3	**0.2**	23.6
	Memory	14.5	248.2	59.0	210.0	**9.8**	46.4
A.thaliana	Time	43.2	8.1	0.6	5.9	**0.2**	12.2
	Memory	10.3	248.2	60.0	210.0	**9.9**	45.3
D.melanogaster	Time	355.2	12.5	0.4	4.8	**0.1**	95.1
	Memory	16.7	204.2	59.0	210.0	**9.7**	48.1

CPU time is in hours and the memory in GB. The best results are in bold

### Sensitivity variations

As we have described above, we use a more precise evaluation compared to the one of Berlin et al. [11]. As a result the programs exhibit a decrease in sensitivity. It is therefore interesting to compare our procedure with the one of Berlin et al. [11]. In Table 4, four ways of evaluating the sensitivity are compared. In our evaluation we check for precise bounds of the alignment; this is given in the rows labelled as “bounds” in the table. We can relax this condition by checking only the length of the alignment; labelled as “length” in the table, this is the closest to the procedure of Berlin et al.. Finally, the weakest check we can have is simply for the “presence” of an alignment between the reads. While there are differences among all these sensitivity modes, HISEA remains clearly the first, followed by DALIGNER and MHAP, and then at some distance by the other three programs. It is interesting to note the very high sensitivity of DALIGNER in the “presence” only scenario.

**Table 4:** Comparison of several types of sensitivity computations on the 1Gbp datasets

For each dataset, four types of sensitivity computations are used: “presence” only checks for the read pair, “length” also checks the correct length, “bounds” checks for correct alignment bounds (the one used in this paper), and the last one is from Berlin et al. [11]

### MHAP sketch size and Minimap minimizers

Both MHAP and Minimap can have their parameters adjust to improve sensitivity. We investigate here this effect.

MHAP uses a technique called MinHash [14] in order to compute the overlaps. MinHash reduces a string to a set of fingerprints, called sketch. It is clear that using a larger sketch increases the sensitivity at the cost of speed decrease. Given the excellent speed of MHAP, it is worth investigating the effect of this parameter. Note that we already tested sketch size 1256 instead of the default 512, for improved sensitivity. Table 5 shows the results for sketch size increased with increments of 512 from 1256 to 3816. The sensitivity increases slightly but never comes close to that of HISEA. Also, precision decreases and so the *F*_1_-score increases very little (or decreases dramatically, as it happens for *C.elegans*). Also, the running time increases up to 10 times when changing sketch size from 1256 to 3816. Overall, increasing the sketch size is clearly not improving the performance of MHAP.

**Table 5:** Testing larger sketch sizes for MHAP. Starting with the value we have used for testing, 1256, the sketch size is increased with increments of 512 up to 3816

Genome	Parameter	MHAP skecth size					
		1256	1768	2280	2792	3304	3816
*E.coli*	Sensitivity	83.74	85.75	86.52	86.87	87.05	87.16
	Specificity	99.90	99.86	99.84	99.82	99.81	99.80
	Precision	97.15	96.99	96.82	96.89	96.88	97.70
	F1-score	89.95	91.02	91.38	91.61	91.70	92.13
*S.cerevisiae*	Sensitivity	62.08	63.67	64.32	64.52	64.62	64.69
	Specificity	99.77	99.72	99.66	99.63	99.58	99.56
	Precision	89.29	88.79	88.69	88.62	88.55	88.30
	F1-score	73.24	74.16	74.56	74.67	74.72	74.67
*C.elegans*	Sensitivity	80.43	81.81	82.37	82.62	82.69	82.73
	Specificity	99.97	99.93	99.90	99.88	99.85	99.82
	Precision	45.46	35.71	29.32	25.80	23.75	22.13
	F1-score	58.09	49.72	43.25	39.32	36.90	34.92
*A.thaliana*	Sensitivity	76.19	77.05	77.38	77.49	77.55	77.57
	Specificity	99.91	99.87	99.86	99.85	99.84	99.83
	Precision	88.78	88.50	88.68	88.35	88.55	88.33
	F1-score	82.00	82.38	82.65	82.56	82.69	82.60
*D.melanogaster*	Sensitivity	71.86	73.36	73.89	74.12	74.24	74.30
	Specificity	99.94	99.92	99.91	99.88	99.87	99.86
	Precision	72.47	72.00	72.07	72.46	71.45	71.62
	F1-score	72.16	72.67	72.97	73.28	72.82	72.94

Note that the results for the first column (sketch size 1256) appear also in Table 2. They are repeated here for comparison convenience

Similarly, the sensitivity of Minimap can be increased by using more minimizers. A minimizer is the smallest *k*-mer in a window of *w* consecutive *k*-mers. The default value is *w*=10 but the recommended value by the designers for all-vs-all PacBio read self-mapping is *w*=5 and this is what we used in our tests. We have investigated the effect of increasing the number of minimizers by decreasing *w*. The results are presented in Table 6. The improvement is more significant for Minimap but it starts from lower values. The improved performance is still far from the top programs.

**Table 6:** Testing higher number of minimizers for Minimap. Starting with the value we have used for testing, *w*=5, we increase the number of minimizers by decreasing *w* all the way to the smallest value *w*=1. Note that the results for the first column (*w*=5) appear also in Table 2. They are repeated here for comparison convenience

Genome	Parameter	Minimap window size				
		5	4	3	2	1
*E.coli*	Sensitivity	91.80	93.08	94.13	95.24	96.29
	Specificity	99.93	99.92	99.93	99.92	99.91
	Precision	97.13	97.22	97.42	97.51	97.58
	F1-score	94.39	95.10	95.75	96.36	96.93
*S.cerevisiae*	Sensitivity	9.35	9.64	9.94	10.36	11.00
	Specificity	99.98	99.98	99.97	99.97	99.97
	Precision	94.30	94.18	93.28	91.90	88.58
	F1-score	17.01	17.49	17.97	18.62	19.57
*C.elegans*	Sensitivity	85.38	86.63	87.63	88.77	89.80
	Specificity	99.98	99.98	99.98	99.98	99.97
	Precision	89.80	89.77	89.05	88.11	85.76
	F1-score	87.53	88.17	88.33	88.44	87.73
*A.thaliana*	Sensitivity	23.55	26.90	31.21	37.08	45.56
	Specificity	99.97	99.98	99.96	99.96	99.96
	Precision	84.00	84.77	85.48	86.43	87.94
	F1-score	36.79	40.84	45.73	51.90	60.02
*D.melanogaster*	Sensitivity	40.72	42.82	45.51	49.11	54.00
	Specificity	99.99	99.98	99.98	99.98	99.97
	Precision	83.93	83.12	82.87	81.85	81.25
	F1-score	54.84	56.52	58.75	61.39	64.88

### Sensitivity vs overlap size

**Fig. 3:**
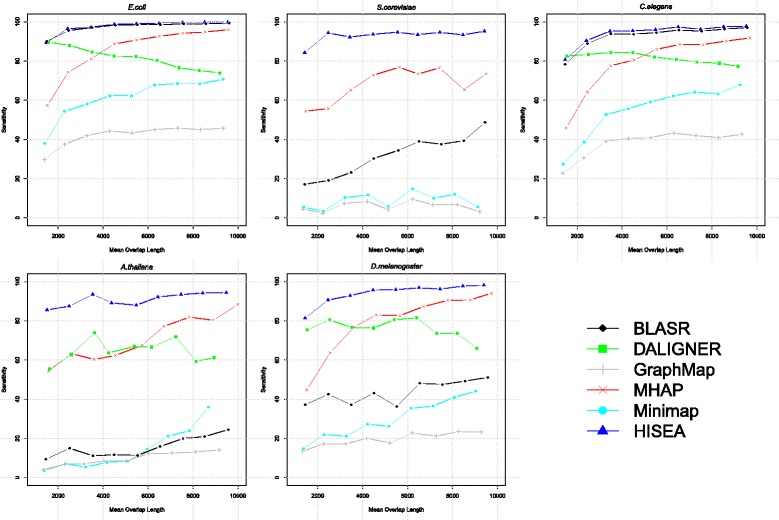
Sensitivity as a function of mean overlap length

It is easier to find long overlaps with correct bounds compared to short overlaps. We have plotted in Fig. 3 the aligners’ sensitivity as a function of overlap length. The sensitivity increases with the overlap length for all aligners except DALIGNER. The sensitivity of HISEA remains very high for both short and long overlaps and it improves with longer overlap lengths. MHAP shows a similar trend but its sensitivity for short overlaps is low. BLASR, Minimap, and GraphMap seem to have been optimized for more recent chemistry; note the very low performance on the oldest chemistry P4C2 datasets.

### HISEA vs MHAP

Since sensitivity is the most important parameter, as long as the difference in precision is not too large, we compare for the remaining tests only the top two programs, HISEA and MHAP. It turns out that the way MHAP is run within the Canu assembly pipeline is different from running it in stand alone mode. Therefore, we are comparing again the sensitivity, specificity and precision of the alignment produced by the two programs, this time while run in the pipeline mode.

We consider the same datasets as above but with higher coverage: 30x and 50x. As mentioned by Koren et al. [6], Canu+MHAP pipeline reaches the best assemblies around 50x coverage. Our goal is to produce similar quality assemblies with only 30x coverage. The 30x and 50x coverage datasets were sampled using the utility *fastqSample* available from the Canu pipeline [6].

The alignments computed by MHAP and HISEA while run in the Canu pipeline were extracted and analyzed as above. The results are shown in Table 7. HISEA has better sensitivity, precision, and *F*_1_ score in all tests with very large differences for the 50x coverage datasets. The specificity of both programs is very high for all tests, with HISEA edging ahead for 30x coverage and MHAP for 50x.

**Table 7:** Sensitivity, specificity, precision, and *F*_1_-score for HISEA and MHAP program output within the Canu pipeline

Two coverage levels are considered for each dataset: 30x and 50x. The best values are shown in bold. The bottom of the table shows the average values, each computed from the five corresponding values in the table. All values are percentages

### Assembly comparison

We have integrated the HISEA program in the Canu assembly pipeline, which is currently the best. Our alignment output is similar to the M4 format used by BLASR and MHAP programs^1^. HISEA can also be integrated in other assembly pipelines, e.g., Miniasm [5] and Falcon [7], by converting HISEA output to the format required by these pipelines.

We have assembled the 30x and 50x coverage datasets that we tested above for quality of alignments. The assemblies produced by the two pipelines, Canu+MHAP and Canu+HISEA, have been evaluated using a modified version of our LASER program [15], which is a fast implementation of QUAST [16] using E-MEM [17]. The recent versions of QUAST use E-MEM [17] for speed improvement but LASER [15] has several other modifications that make it still faster.

LASER/QUAST compute many parameters for each assembly and the most important ones are presented in Table 8: the number of contigs, NG50, the maximum contig size, the fraction of the genome covered by the assembly, the identity with the reference, and the number of breakpoints (inversions, relocations, and translocations). The Canu+HISEA pipeline has better values in 80% of the tests for the number of contigs, NG50, max contig size, and genome fraction. Generally, the NG50 value for the Canu+HISEA assemblies is much larger than that of the Canu+MHAP ones. Canu+MHAP has fewer breakpoints more often than Canu+HISEA but the difference is usually small. Both pipelines have high identity with the reference. Overall, the assemblies computed by the Canu+HISEA pipeline are better. Moreover, the assemblies computed by Canu+HISEA for 30x coverage are comparable with those produced by Canu+MHAP for 50x coverage. MUMmer plots of all Canu+HISEA assemblies are included in the Additional file 1.

**Table 8:** Pipeline assembly comparison; Canu assembler is used with MHAP and HISEA as read aligners

Genome	Parameter	Canu + MHAP		Canu + HISEA	
		30x	50x	30x	50x
*E.coli*	Contig #	**7**	3	8	**1**
	NG50	**2,771,323**	3,969,196	1,223,211	**4,642,165**
	Max contig	**2,771,323**	3,969,196	1,525,215	**4,642,165**
	% Ref	**99.85**	99.97	99.82	**100.00**
	Avg idy	**99.97**	**99.99**	**99.97**	**99.99**
	Breakpoints	**3**	**3**	**3**	**3**
*S.cerevisiae*	Contig #	43	31	**35**	**29**
	NG50	540,299	687,498	**682,168**	**774,485**
	Max contig	964,505	1,534,125	**1,537,586**	**1,534,133**
	% Ref	98.90	99.35	**99.12**	**99.58**
	Avg idy	99.81	**99.88**	**99.82**	**99.88**
	Breakpoints	**17**	**14**	**17**	**14**
*C.elegans*	Contig #	393	170	**127**	**133**
	NG50	636,401	1,987,017	**2,140,282**	**2,032,954**
	Max contig	2,648,207	4,224,025	**4,227,561**	**5,669,072**
	% Ref	96.00	**99.84**	**99.81**	99.80
	Avg idy	99.76	**99.91**	**99.85**	**99.91**
	Breakpoints	431	**423**	**390**	435
*A.thaliana*	Contig #	159	**99**	**140**	122
	NG50	3,331,858	6,715,370	**5,069,662**	**8,124,422**
	Max contig	**12,892,206**	14,177,369	12,890,806	**15,940,320**
	% Ref	92.22	**92.55**	**92.37**	92.51
	Avg idy	**99.17**	**99.22**	**99.17**	**99.22**
	Breakpoints	**2,550**	**2,693**	2,680	2,704
*D.melanogaster*	Contig #	597	390	**553**	**372**
	NG50	1,933,939	4,983,913	**6,417,268**	**13,672,005**
	Max contig	8,238,062	17,900,724	**17,366,974**	**25,767,672**
	% Ref	95.08	98.55	**96.47**	**98.65**
	Avg idy	**99.80**	**99.89**	**99.80**	99.87
	Breakpoints	**1,039**	**1,383**	1,254	1,461

Two coverage levels, 30x and 50x, are used for each genome. The best results are shown in bold

The MHAP program is very fast and it makes the Canu+MHAP pipeline faster, as seen from the time values shown in Table 9. However, as noticed above, similar assemblies are produced by Canu+HISEA for 30x coverage, and those are always faster than those by Canu+MHAP for 50x coverage. The memory consumption is always much lower for the Canu+HISEA pipeline. Note that in Table 9 the times are reported as wall clock times, since CPU times for the fraction used by the overlapping programs are not available. Also, only the peak memory used by the entire assembly pipeline is available.

**Table 9:** Assembly time and space comparison; the time is wall clock time in hours, the space is in GB

Genome	Canu + MHAP				MHAP		Canu + HISEA				HISEA	
	30x		50x		30x	50x	30x		50x		30x	50x
	Time	Space	Time	Space	Time	Time	Time	Space	Time	Space	Time	Time
*E.coli*	**0.4**	210	**0.6**	210	**0.1**	**0.1**	**0.4**	**25**	0.7	**40**	**0.1**	**0.1**
*S.cerevisiae*	**1.1**	210	**2.0**	210	0.3	**0.4**	1.2	**63**	2.9	**76**	0.2	0.6
*C.elegans*	**24.5**	210	**59.6**	210	**2.4**	**2.5**	37.7	**83**	75.5	**82**	11.5	17.1
*A.thaliana*	**23.8**	210	**56.6**	210	**4.1**	**9.6**	42.3	**90**	98.0	**90**	15.3	35.0
*D.melanogaster*	**27.0**	210	**62.4**	210	**3.4**	**5.2**	51.8	**94**	112.8	**94**	19.7	33.6

The same setup as in Table 8 is used. The best values are in bold

The *java* command-line parameter *-Xmx* is used to set the maximum heap size during MHAP invocation from the pipeline. The value of parameter *-Xmx* is set by *corMhapMemory* pipeline parameter which is user configurable. For this evaluation, the value of parameter *corMhapMemory* is set to 200 Gb for all datasets. The peak memory in each case is reported as 210 Gb. Similar configuration for Canu+HISEA pipeline uses much smaller memory footprint (less than 100 Gb) for all datasets.

The Canu+MHAP pipeline requires more memory in all cases, as seen from the space values shown in Table 9. The peak memory of this pipeline can be reduced by setting a smaller value for *corMhapMemory*. However, it impacts the overall assembly runtime. Similar behavior is expected in modified Canu+HISEA pipeline. To ensure unbiased evaluation, all parameter values are identical for both pipelines.

## Discussion

The newly introduced HISEA program has been thoroughly tested against several state-of-the-art programs and shown to perform better. HISEA has higher sensitivity, precision, and F1-score. Two competing programs, MHAP and Minimap, have parameters that can be tuned for a trade-off between speed and sensitivity. We pushed both to the limit of their sensitivity and that is still clearly lower than the sensitivity of HISEA. Since we introduced a new, more precise, evaluation of sensitivity, we compared also the programs with respect to the old method of computing sensitivity, as well as two other natural ways. HISEA has the highest sensitivity with respect to all four sensitivity modes. The closest competitor is MHAP and we compared the two programs further, from the point of view of genome assembly. HISEA is significantly more sensitive and produces better genome assemblies in the Canu pipeline.

## Conclusion

Pacific Biosciences SMRT technology is a relatively new sequencing method that produces long but noisy reads. The aligners developed for previous sequencing methods do not perform well on this type of data. Our new HISEA algorithm for computing read alignments has introduced several new ideas, such as clustering of *k*-mer matches, estimating and filtering of matches based on error rate, and techniques for extending the alignments with shorter *k*-mer matches.

The HISEA algorithm currently produces alignments with highest sensitivity and comparable specificity with other algorithms. Integrated in the Canu pipeline [6], currently the best for assembling PacBio data, it produces better assemblies than Canu+MHAP. Moreover, the assemblies of Canu+HISEA at lower coverage, 30x, are comparable with those of Canu+MHAP at 50x coverage, while being faster and cheaper. We plan to modify HISEA in the future to work also with Oxford Nanopore sequencing technology [18]. The source code of the HISEA aligner and Canu+HISEA assembly pipeline are freely available from: https://github.com/lucian-ilie/HISEA and https://github.com/lucian-ilie/Canu_HISEA, respectively.

## Additional file

Additional file 1 The additional material contains information concerning downloading the datasets, the versions used for each competing program, download websites, and command lines. (PDF 412 kb)

## Footnotes

1
https://github.com/PacificBiosciences/blasr/wiki/Blasr-Output-Format
