# Supplementary material for: HISEA: HIerarchical SEed Aligner for PacBio data
Source: BMC Bioinformatics. 2017 Dec 19;18:564. doi: 10.1186/s12859-017-1953-9 (PMC5735879; doi:10.1186/s12859-017-1953-9)
Supplement: Additional file 1 — The additional material contains information concerning downloading the datasets, the versions used for each competing program, download websites, and command lines. (PDF 412 kb) [file 12859_2017_1953_MOESM1_ESM.pdf]

# HISEA: HIerarchical SEed Aligner for PacBio data

Nilesh Khiste and Lucian Ilie

– Supplementary material –

## 1 Sub-sampled Genome Sequences

The datasets used for this evaluation are mentioned in HISEA paper. All the datasets have been downloaded from Pacific Biosciences DevNet Datasets (https://github.com/PacificBiosciences/DevNet/wiki/Datasets) website. The 30X and 50X sub-sampled datasets used for this evaluation were extracted using the utility *fastqSample* available from the Canu pipeline.

## 2 Programs

### 2.1 Download details

We provide details on the versions used for each program. SHA1 values for each zip files are also provided (as computed using the Unix/Linux utility sha1sum).

1. BLASR - downloaded on May 26, 2015
   https://github.com/PacificBiosciences/blasr
   sha1: b9de0d897c5eb96da43a2806420bef9e42d3e644 master.zip

2. DALIGNER downloaded on May 17, 2016
   https://github.com/thegenemyers/DALIGNER
   https://github.com/thegenemyers/DAZZ_DB
   sha1: 8f8a31e7855a285c93169befa1bcd7a77235b90e daligner.zip
   sha1: 45d2b30383f28c4c6c5809e38e83c3e137323af0 db.zip

3. MHAP - version 2.1, downloaded on August 10, 2016
   https://github.com/marbl/MHAP
   sha1: e50bbe13ce4679c16f59ddc830c4dae0d28f32cd v2.1.tar.gz

4. Minimap - version 0.2-r124-dirty, downloaded on October 8, 2016
   https://github.com/lh3/minimap
   sha1: bbc5b512656805edc1b8b290612d3d21bffebe67 master.zip

5. GraphMap - version: v0.5.0, downloaded on March 2, 2017
   https://github.com/isovic/graphmap
   sha1: b2674360f706c5a64aa873bbaa4a2dfb69a08d11 master.zip

6. Canu - version 1.3, downloaded on July 21, 2016
   https://github.com/marbl/canu/releases
   sha1: 523f7bcb147dfd39b92b3e5704ba2f1262d0edc2 v1.3.tar.gz

The sha1 values for HISEA and new Canu pipeline with HISEA support are as given

1. HISEA
   sha1: e40d0ade9d25625d0d9e8218f2e2dede656ac421 HISEA.zip

2. Canu+HISEA
   sha1: 78af7d159a55eeb505207aa187f8d2399a34a03e canu_hisea-1.3.zip

## 2.2 Command-line options

The details of the command-line options used for each program is provided here. The parameter not mentioned here use their default values. The BLASR command-line options have been modified for each datasets to get best possible alignments. DALIGNER requires invocation of multiple binaries such as *fasta2DB, DBDust, daligner, LASort, LAmerge etc.* Binaries are invoked with default parameters or with additional/best options depending on information available on tool usage in documentation. The best options for BLASR and MHAP are used as discussed in MHAP paper. Minimap parameters are used based on the information recommended on their github page. GraphMap has no impact of parameters when run in **owler** mode. Further, GraphMap github page suggests that default parameters work well for all types of sequencing data.

### 2.2.1 Alignment

1. BLASR

   - Escherichia coli K12: -maxScore 1000 -maxLCPLength 16 -minMatch 12 -nCandidates 1000
   - Saccharomyces cerevisiae: -maxScore 1000 -maxLCPLength 16 -minMatch 12 -nCandidates 850
   - Caenorhabditis elegans: -maxScore 1000 -maxLCPLength 16 -minMatch 12 -nCandidates 850
   - Arabidopsis thaliana Ler-0: -maxScore 1000 -maxLCPLength 16 -minMatch 12 -nCandidates 80
   - Drosophila melanogaster: -maxScore 1000 -maxLCPLength 16 -minMatch 12 -nCandidates 80

2. DALIGNER: -v

3. MHAP: –num-hashes 1256
4. Minimap: -Sw5 -L100 -m0
5. GraphMap: owler
6. HISEA: –self –kmerLen 16

#### 2.2.2 MHAP sketch size and Minimap minimizers options

It was suggested that MHAP sensitivity can be significantly improved by increasing sketch-size. We tested MHAP by increasing sketch-size in blocks of 512 hashes. Our results indicate that there is a small increase in sensitivity at the cost of specificity, precision and time (See paper for results). The tests were conducted by using sketch-size ( –num-hashes option) of 1256, 1768, 2280, 2792, 3304 and 3816.

Similar to MHAP, Minimap's sensitivity can be increased by decreasing minimizer window size parameter $w$. Our test indicate that there is an improvement in sensitivity when minimizer size is decreased but the tool is still lagging behind due to lower sensitivity. The tests were conducted for decreasing values of $w = 5, 4, 3, 2$ and 1. For detailed analysis and results, please see main paper.

#### 2.2.3 Assembly

The assembly was generated with identical configuration files for both Canu+MHAP and Canu+HISEA. The configuration files used for HISEA can be downloaded from `https://github.com/lucian-ilie/Canu_HISEA` website.

#### 2.2.4 Alignment Evaluation

The sensitivity and specificity of all the programs have been computed using a modified version of *EstimateROC* utility from MHAP program. The commandline used for this utility is:

*EstimateROC <reference mappings in M4> <program output in M4> <fasta of reads> 2000 50000 false false*

where description of each parameter is as given below:

1. *reference mappings in M4*: The mapping of reads with reference computed using BLASR
2. *program output in M4*: Output of each program in M4 format
3. *fasta of reads*: Reads in fasta format
4. *2000*: Minimum overlap length to be considered
5. *false*: Use dynamic programming for PPV computation
6. *false*: Verbose mode

### 2.2.5 Assembly Evaluation

The assembly generated with Canu+MHAP and Canu+HISEA pipelines were evaluated using LASER, a performance improved version of QUAST program version 4.3. For all the datasets, the LASER program was invoked with following options:

*quast.py <asm.fasta> -o <outputDir> -R <reference.fasta> –gage*

# 3 Plots

The MUMmer plots for the aseemblies produced by the Canu+HISEA pipeline for the 30x and 50x coverage datasets for our five genomes are shown in Figs 1-5. All plots are generated with following set of commands:

*nucmer -mumref -l 100 -c 1000 -d 10 -banded -D 5 <ref.fasta> <asm.fasta>*

*delta-filter -i 95 -o 95 out.delta > out.best.delta*

*mummerplot –fat out.best.delta*

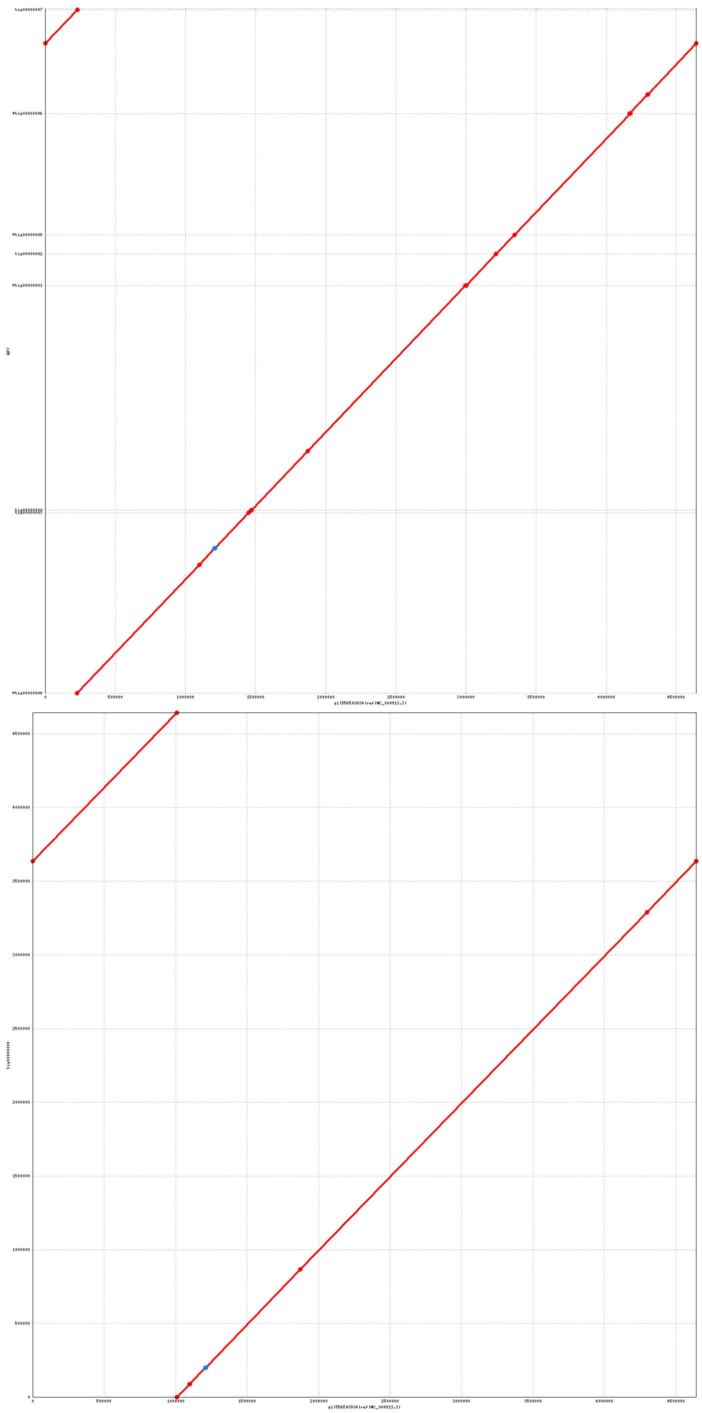

Figure 1: MUMmer plot for Canu+HISEA assembly of *E.coli* 30x (top) and 50x (bottom).

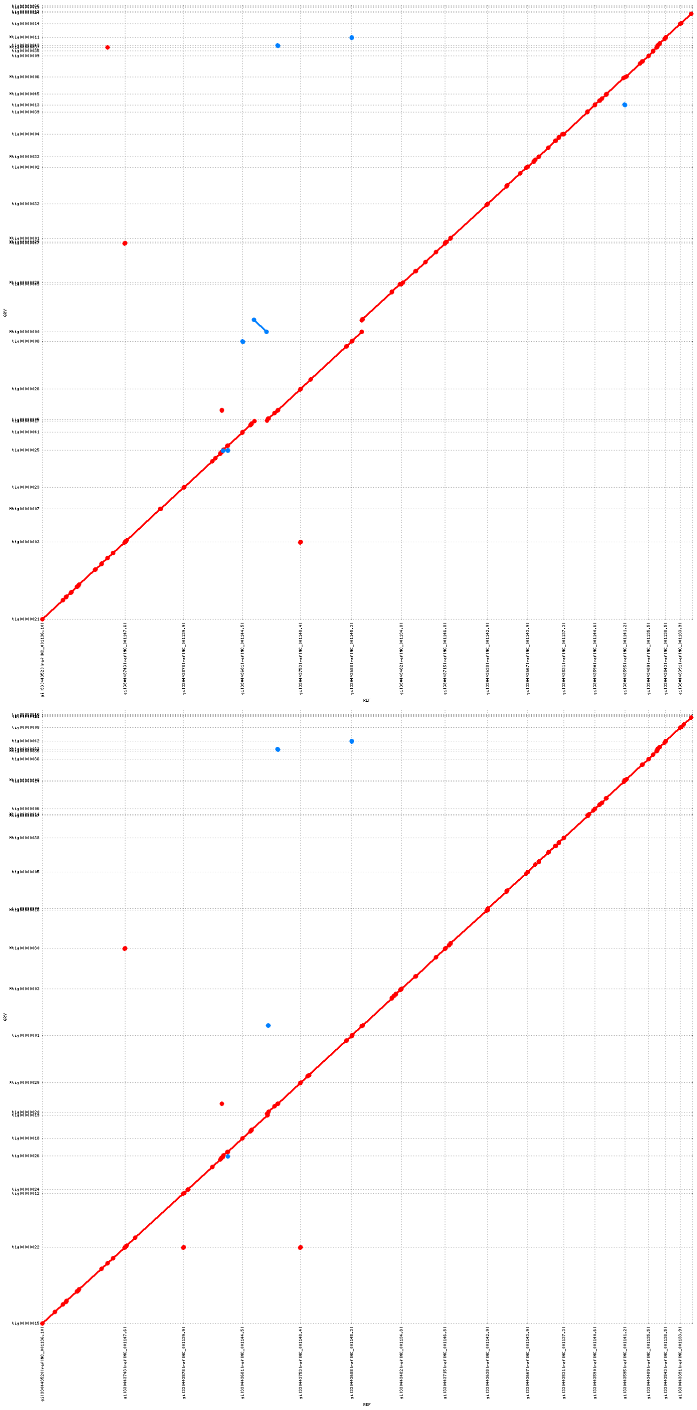

Figure 2: MUMmer plot for Canu+HISEA assembly of *S.cerevisiae* 30x (top) and 50x (bottom).

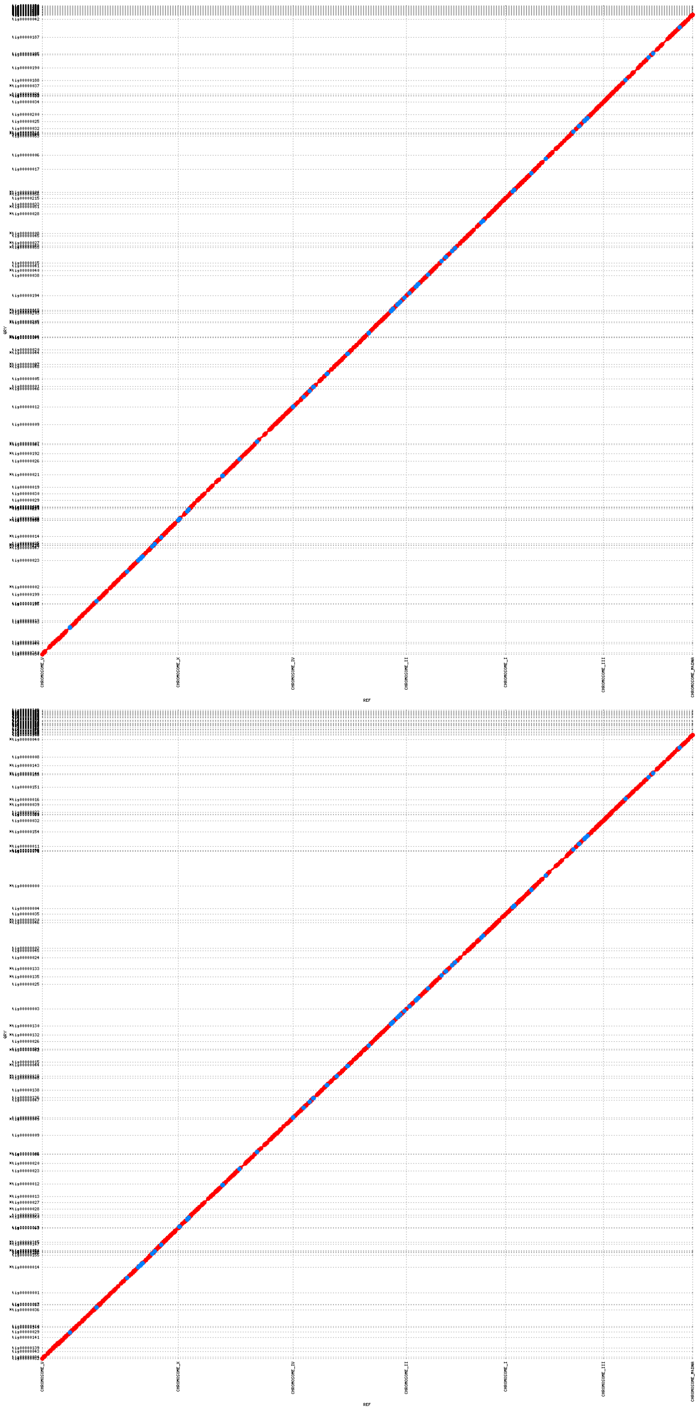

Figure 3: MUMmer plot for Canu+HISEA assembly of *C.elegans* 30x (top) and 50x (bottom).

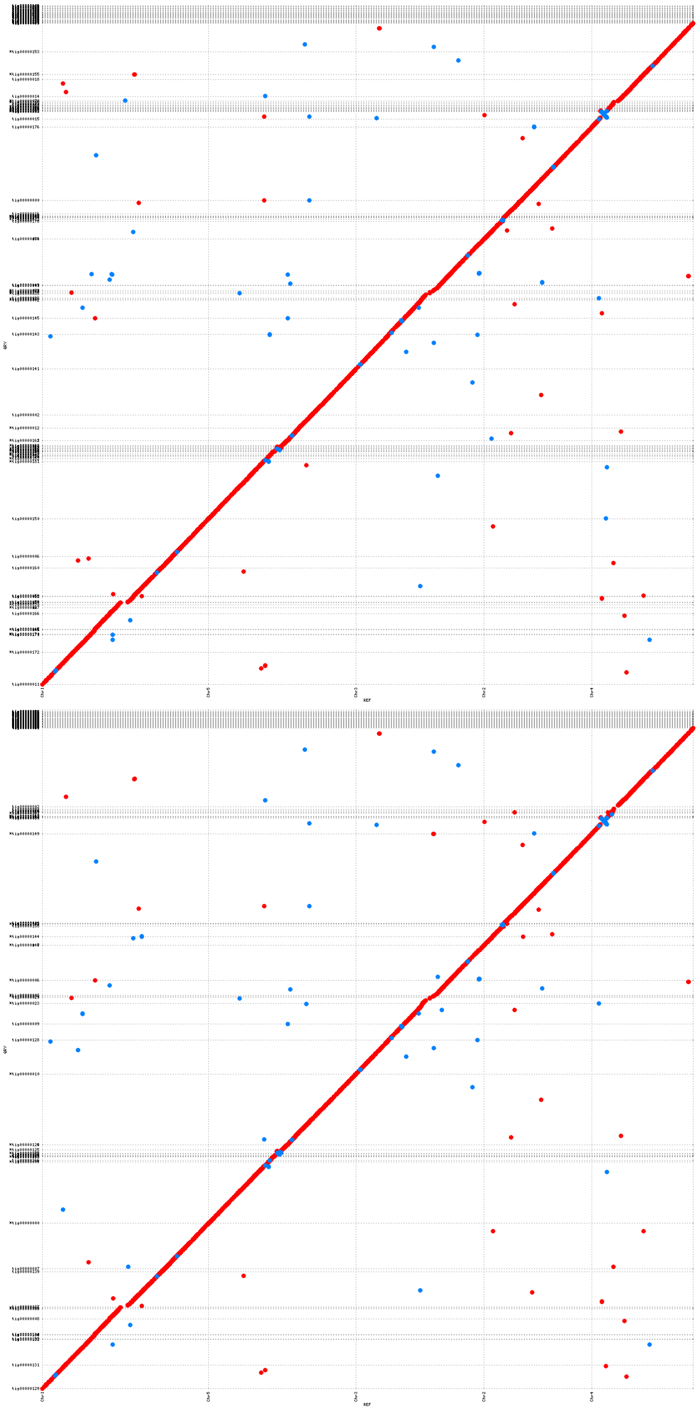

Figure 4: MUMmer plot for Canu+HISEA assembly of *A.thaliana* 30x (top) and 50x (bottom).

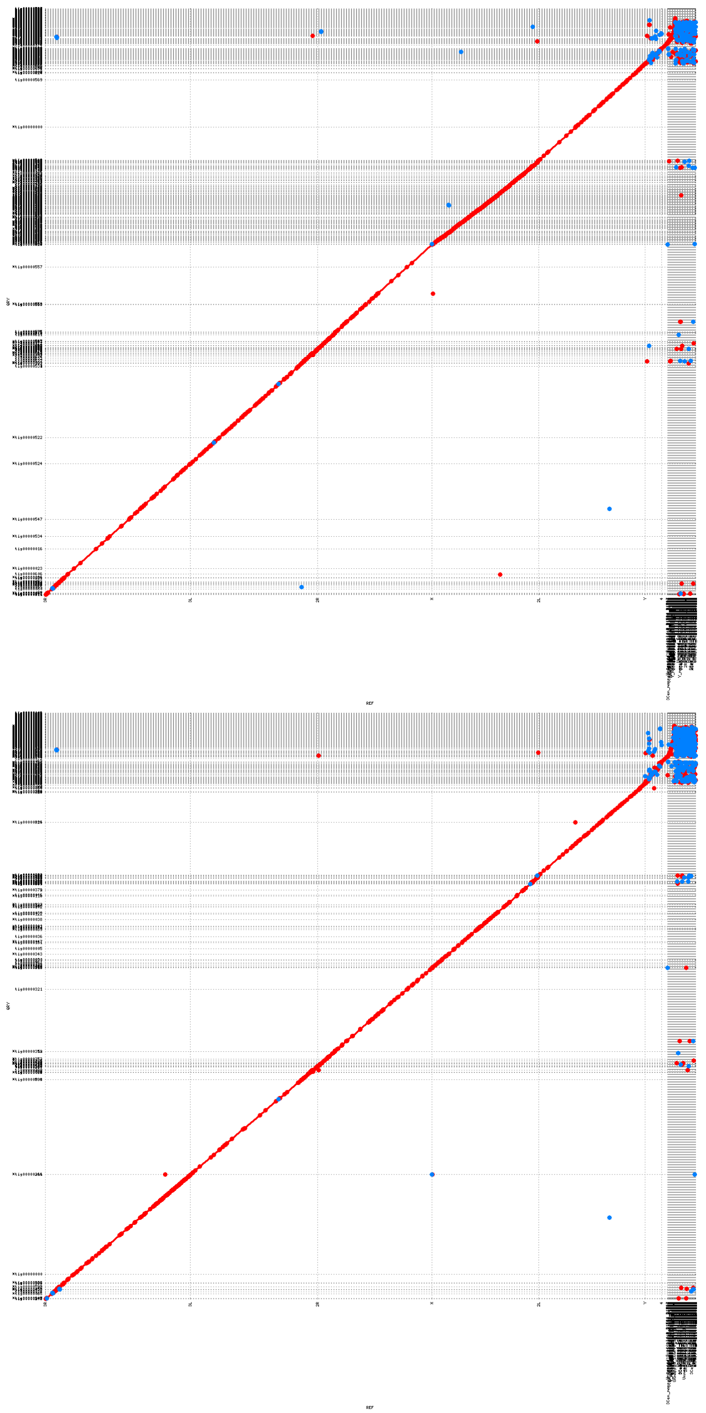

Figure 5: MUMmer plot for Canu+HISEA assembly of *D.melanogaster* 30x (top) and 50x (bottom).
